# Microplastics and Their Impact on Reproduction—Can we Learn From the *C. elegans* Model?

**DOI:** 10.3389/ftox.2022.748912

**Published:** 2022-03-24

**Authors:** Elysia Jewett, Gareth Arnott, Lisa Connolly, Nandini Vasudevan, Eva Kevei

**Affiliations:** ^1^ School of Biological Sciences, University of Reading, Reading, United Kingdom; ^2^ The Institute for Global Food Security, School of Biological Sciences, Queen’s University Belfast, Northern Ireland, United Kingdom

**Keywords:** microplastic particles, reproduction, fertility, *C. elegans* model, ROS, nuclear hormone signaling

## Abstract

Biologically active environmental pollutants have significant impact on ecosystems, wildlife, and human health. Microplastic (MP) and nanoplastic (NP) particles are pollutants that are present in the terrestrial and aquatic ecosystems at virtually every level of the food chain. Moreover, recently, airborne microplastic particles have been shown to reach and potentially damage respiratory systems. Microplastics and nanoplastics have been shown to cause increased oxidative stress, inflammation, altered metabolism leading to cellular damage, which ultimately affects tissue and organismal homeostasis in numerous animal species and human cells. However, the full impact of these plastic particles on living organisms is not completely understood. The ability of MPs/NPs to carry contaminants, toxic chemicals, pesticides, and bioactive compounds, such as endocrine disrupting chemicals, present an additional risk to animal and human health. This review will discusses the current knowledge on pathways by which microplastic and nanoplastic particles impact reproduction and reproductive behaviors from the level of the whole organism down to plastics-induced cellular defects, while also identifying gaps in current knowledge regarding mechanisms of action. Furthermore, we suggest that the nematode *Caenorhabditis elegans* provides an advantageous high-throughput model system for determining the effect of plastic particles on animal reproduction, using reproductive behavioral end points and cellular readouts.

## Microplastics and Nanoplastics Pose Health Risks for Animals and Humans

Plastics (long polymer chains) are widely used due to their versatility and durability, which has led to the accumulation of substantial plastic waste in the environment (MacLeod et al., 2021). The most common plastic polymers found in the environment are polyethylene (PE), polystyrene (PS), polypropylene (PP), polyethylene terephthalate (PET), and polyvinyl chloride (PVC) (Bratovcic, 2019). Macroplastics (1 cm and larger) present ecological problems due to entrapment and entanglement, digestive tract congestion, and physical barriers for food supply (Chapron et al., 2018; Gündoğdu and İYeşilyurtErbaş, 2019). Plastic polymers could be also transformed in size (macro-, micro-, and nanoplastics) and in shape (spheres, fibers, and fragments) upon exposure to UV light, heat, or waves in the aquatic environment, or by biological degradation. These processes lead to environmental weathering of MPs/NPs, which, similarly to aging of plastic particles (Liu et al., 2020a), enhances the leaching of chemicals from these pollutants (Yousif and Haddad, 2013). Endocrine disrupting chemicals (EDCs) used as additives to create these plastics, such as the estrogenic and anti-estrogenic phthalates, polychlorinated biphenyls, and bisphenol A, also interfere with the biology of animals and humans, (Campanale et al., 2020a; Darbre, 2020). Furthermore, because of their large surface area to volume ratio, MPs and NPs can absorb various environmental pollutants, such as polycyclic aromatic hydrocarbons (PAHs), which also act as EDCs (Zhang et al., 2016; Lara et al., 2021), or hydrophobic persistent organic pollutants (POPs), pesticides, heavy metals, and microorganisms (Frias et al., 2010; Curren and Leong, 2019; Yu et al., 2019; Liu et al., 2021a; Coffin et al., 2018), all of which could further aggravate the toxicity of plastics particles.

Nanoplastic particles and microplastic particles, which are less than 100 nm, or less than 5 mm in diameter, respectively, have been found in sewage, soil, oceans, seafood, drinking water, and even table salts (Mason et al., 2018; Hartmann et al., 2019; Lee et al., 2019). Since MPs are too small to be removed by sewage filtration, they can wash into the sea where they accumulate in most bodies of water. MPs have unique properties which can facilitate internalization by biota. They provide visual stimulus for ingestion by animal species (Carpenter et al., 1972; Gramentz, 1988; David and Robert, 1994), or chemical cues for other foragers for preferential ingestion of MP-containing food (Savoca et al., 2016; Savoca et al., 2017; Savoca et al., 2018; Procter et al., 2019). Accumulation of MPs and NPs have been widely recorded in various aquatic (Lusher et al., 2013; Avio et al., 2015; Frydkjær et al., 2017; Gambardella et al., 2017; Critchell and Hoogenboom, 2018; Lo and Chan, 2018; Naidoo and Glassom, 2019; Masiá et al., 2021; Stienbarger et al., 2021; Liu et al., 2022a) (reviewed in: (Akdogan and Guven, 2019; Wang et al., 2019a; Franzellitti et al., 2019)) and terrestrial animals (Huerta Lwanga et al., 2017; Maaß et al., 2017; Souza Machado et al., 2018; Panebianco et al., 2019; Lu et al., 2020; Mackenzie and Vladimirova, 2021). These studies have reported significant detrimental effects on animal development and health, including intestinal defects, decreased body size, decreased survival rate and reproduction, decreased motility, altered behavior, neurotoxicity, increased inflammation, oxidative stress, genotoxicity, altered fat and energy metabolism, and changes in the microbiome (Tosetto et al., 2016a; Lu et al., 2016; Lei et al., 2018a; Jin et al., 2018; Fackelmann and Sommer, 2019; Poma et al., 2019; Qiao et al., 2019; Li et al., 2020a; Araújo and Malafaia, 2020; Crump et al., 2020; Hirt and Body-Malapel, 2020; Prüst et al., 2020; Solleiro-Villavicencio et al., 2020; Yong et al., 2020; Li et al., 2021a; Lear et al., 2021; Tagorti and Kaya, 2022). MPs and NPs also pose health risks for humans. MPs and NPs are taken up through inhalation, ingestion and via skin contact (Leslie, 2014; Gasperi et al., 2018; Pivokonsky et al., 2018; Prata, 2018; Hantoro et al., 2019; Koelmans et al., 2019; Toussaint et al., 2019; Vianello et al., 2019; Campanale et al., 2020b; Danopoulos et al., 2020; Prata et al., 2020; Rahman et al., 2021; Senathirajah et al., 2021; Vethaak and Legler, 2021), and these plastic particles have been found in the human lung (Pauly et al., 1998; Vianello et al., 2019), intestine (Schwabl et al., 2019) and placenta (Ragusa et al., 2021). Recently, NPs have been shown to be transmitted to offspring of NP-exposed zebrafish mothers (Wang et al., 2019a), suggesting that MPs and NPs have an impact on the health of multiple generations of animals and potentially humans (Pitt et al., 2018; Ragusa et al., 2021). This review aims to detail common effects of MPs/NPs on reproduction compared across several model organisms and provide evidence that *C. elegans* is an advantageous model to study the effects of MPs/NPs on animal health.

## Reproductive Effects of Microplastics and Nanoplastics Exposure

Fertility is the ability to produce offspring and is critically dependent on gonad tissue integrity, as well as egg and sperm quality. In aquatic models such as Brine shrimp (*Artemia franciscana*) (Gambardella et al., 2017), the water flea (*Daphnia magna*) (An et al., 2021a; Liu et al., 2021b; Trotter et al., 2021), the pacific oyster (*Crassostrea gigas*) (Sussarellu et al., 2016; Tallec et al., 2021), marine medaka (*Oryzias melastigma*) (Chisada et al., 2021; Wang et al., 2021), sea urchins (*Sphaerechinus granularis*) (Gambardella et al., 2018), marine copepods (Cole et al., 2015; Heindler et al., 2017; Zhang et al., 2019; Yu et al., 2020a), and zebrafish (*Danio rerio*) (Sarasamma et al., 2020; Qiang and Cheng, 2021), MP and NP-induced reproductive toxicity is represented by production of fewer offspring or clutch, lower number of spawned eggs per clutches, increased interval between clutches, or the presence of lower number of gravid females (Supplementary Tables S1, S2). In the following sections we discuss some common effects of plastic particles from studies where reproductive toxicity was shown upon MP or NP exposure. We also provide an overview of how *C. elegans* mechanistic studies can advance our knowledge on plastic-mediated reproductive toxicity.

## Characteristics of MPs and NPs That Cause Reproductive Toxicity

### The Impact of Size, Shape or Chemical Composition of Plastic Particles on Reproductive Toxicity

Researchers have looked at the impact of a large size range of NPs/MPs and tested the effects of various plastic types and shapes in a wide variety of animal species. As shown in Supplementary Tables S1, S2, it is clear that in most cases small MPs are more toxic than larger ones. For example, PS-MPs sized from 0.05 µm (NP) to 6 µm (MP) applied to marine medaka larvae led to decreased hatching rate, with the lowest values observed upon the smallest particle exposure. Paradoxically, this smallest sized NP induced higher expression level of the low choriolytic enzyme (LCE) (Chen et al., 2020a), a hatching enzyme, which could be a compensatory mechanism to counteract MP/NP induced reproductive inhibition. Similarly, in the pacific oyster, PS-NP (50 nm) reduced gamete fertilization, larval development, and embryo hatching, and this occurred regardless whether or not the PS-NPs were amino or carboxyl modified. On the contrary, PS-MPs of 2 µm had no effect on oyster reproduction (Tallec et al., 2018). A similar study using 50 nm amino-modified PS-NP showed no effect on reproduction in the oyster at lower NP concentrations, while higher concentrations of plastic particles reduced sperm motility due to sperm aggregation (Tallec et al., 2020), suggesting that concentration is a critical parameter in MP/NP-induced reproductive toxicity. In marine rotifer species, PS-NPs increased reproductive time and led to oxidative stress to a greater extent that PS-MPs did (Jeong et al., 2016). Exposure to smaller PE-MPs resulted in lower numbers of broods per female in the water flea (*Daphnia magna*), when compared to exposure to larger PE-MPs (Ogonowski et al., 2016). In *C. elegans* hermaphrodites 20 nm NPs caused greater transgenerational oxidative stress with greater induction of stress-responsive genes in the offspring of treated mothers than 100 nm NPs (Liu et al., 2021c), indicating that the smaller the NPs size is, the greater the observed reproductive defects are. Microplastic fibers were typically more toxic than beads. In the amphipod, *Hyalelia azteca*, and in the water flea a greater decrease in reproduction was observed with lower number of broods at lower MP concentrations upon fiber exposure than with plastic beads (Au et al., 2015; Ziajahromi et al., 2017). In the earthworm (*Lumbricus terrestris*) (Huerta-Lwanga et al., 2021) and springtail (*Folsomia candida*), microfibers decreased reproduction by inhibiting spermatogenesis (Jemec Kokalj et al., 2021). In the earthworm *Eisenia andreii*, PE-MP breakdown into NP induced sperm damage and decreased number of sperm bundles but did not cause damage to the oocyte in females, showing sexually dimorphic reproductive toxicity (Jeong et al., 2021). These data suggest that smaller particles are more toxic than larger ones, independent of the chemical composition of the MPs/NPs.

Smaller particles may be toxic because they might be preferentially ingested and thereby decrease ingestion of food. In the marine copepod, PS-MPs were preferred to food, and this led to decreased ingestion of food and increased time to egg hatching (Li et al., 2020b). However, MP ingestion does not preferentially occur if there is an excess of natural food, as can be seen with *Daphnia magna* (Aljaibachi and Callaghan, 2018), marine medaka (Cong et al., 2019), and marine rotifers (Xue et al., 2021). This suggests that active avoidance of plastic particles is possible, though this phenomenon appears to be reported in aquatic rather than in terrestrial species. When *C. elegans* was exposed to PS-MPs reproductive toxicity has been observed, even though the plastic particles were not detected in the reproductive tissues. However, reproductive toxicity were not due to styrene monomers leaching from the beads as their levels used in the study were far below toxicity and PS-MPs do not have to be ingested to have a toxic effect on the worms (Mueller et al., 2020). PS-MPs might indirectly affect reproduction in *C. elegans*, limiting food availability, as is suggested for copepods (Cole et al., 2015), since the inhibitory effects of PS increased with decreasing bacterial densities. In *C. elegans*, PS-MPs up to 3 µm could be ingested, but all sizes from 0.1 to 10 µm decreased the number of offspring. Indeed, the reproductive toxicity correlated with decreased food ingestion (Mueller et al., 2020), suggesting that the presence of PS-MPs interfered with feeding. In the pearl oyster and the planktonic doliolid, decreased feeding and lower ingestion of MPs have been observed instead (Gardon et al., 2018; Paffenhöfer and Köster, 2020); this led to gamete apoptosis to conserve energy for survival in the pearl oyster (Gardon et al., 2018). Smaller sizes of plastics may be more toxic due to longer periods of action, staying in the gut for a longer period of time (Mueller et al., 2020), or easier and preferential ingestion. Furthermore, when ingested, smaller particles could be taken up more easily by cells, using the cellular endocytic machinery or phagocytosis (Rejman et al., 2004; Xia et al., 2008; Ekkapongpisit et al., 2012; Monti et al., 2015). This could lead to cellular internalization and translocation of NPs from exposure site to distant tissues (Rubio et al., 2020).

Combining MPs with other pollutants could also alter the effect of MPs on reproduction due to change in particle size. For example, aggregation of PS-MPs caused by dibutyl phthalate (DBP) led to the formation of very large size particles which could not be ingested by the marine copepod. Hence, the presence of DBP decreased reproductive toxicity of PS-MP, measured as time to hatch, while PS-MP absorbed DBP and decreased DBP toxicity (Li et al., 2020b).

### Surface Modification of MPs or NPs Could Affect Their Toxicity

In *C. elegans* hermaphrodites, a study utilizing unmodified and amino-modified polystyrene NPs revealed that amino-modified NPs were more toxic to reproduction at both 10 μg/L and 100 μg/L concentrations across multiple (F0–F3) offspring generations. Amino modified NPs caused greater and dose dependent reduction in the number of germline cells, fertilized eggs and overall brood size, than pristine, unmodified NPs. The germline defects were due to an upregulation of the pro-apoptotic *ced-3* and *ced-4* genes and a concomitant decrease in the anti-apoptotic *ced-9* gene expression (Sun et al., 2021). Overall, positively charged amino-modified nanoplastics were more toxic in *C. elegans* than neutral NPs, which however were more toxic than the negatively charged carboxylated NPs, possibly due to differential interaction of these compounds with membranes and organelles (Schultz et al., 2021). However, at short-term exposure (i.e., 24 h) and using polystyrene MPs rather than NPs, decreased number of progeny was seen independent of surface modification, although neutral PS particles had larger impact than amino- or carboxy-modified particles on another MP-altered pathway, purine metabolism (Kim et al., 2020). This reinforces the idea that in case of smaller sized particles, such as NPs, amino-modified plastic particles are the most toxic under chronic exposure.

### Plastic Particles of Various Chemical Properties Cause Reproductive Defects

In *Daphnia magna*, exposure to various doses (10–500 mg/L) of MPs (<60 µm) over 21 days revealed the greatest reduction in the number of offspring in the PVC MP treated group, when compared to polyurethane and polylactic acid particles (Zimmermann et al., 2020). However, in *C. elegans* and zebrafish, a comparison of PS, PVC, poly (p-phenylene oxide) (PPE), polyamide (PA) MPs at very low doses (0.001–10 mg/L) and sizes (0.5, 1, 10 μM) showed decreased growth and reproduction rate, independent of dose and chemical property of the particles applied. MP exposure caused intestinal damage and increased gluthathione-S-transferase (GST) levels in a particle size dependent manner (Lei et al., 2018a). PS-MPs also decreased sperm fertilization rate in the sea urchin to a greater extent than polymethyl-methylacrylate (PMMA) particles (Trifuoggi et al., 2019). In general, PS-MPs appear to be more reproductively toxic that other MPs in both aquatic species and in *C. elegans*, although this might be a consequence of most studies using PS particles and the general lack of comparative studies.

### Combinations of MPs or NPs With Other Pollutants Could Aggravate Toxicity on Reproduction

When investigating the impact of MPs/NPs on living organisms, we need to consider that bioactive compounds are almost always present on and readily released from plastic particles. MPs and NPs can carry various toxic pollutants, however, whether these act synergistically, additively or have no impact on effects of MPs/NPs is currently a controversial topic, due to the use of a diverse range of animal species, types of MPs/NPs and pollutants, as well as assessing various physiological or molecular readouts. Some reports indicate that MPs/NPs and their leached EDCs modify each other’s effects on animal survival, reproduction, stress or other signaling pathways, while others report the lack of these (Eder et al., 2021). For example, the pesticide deltamethrin caused delayed first brood production and decreased fertility in *Daphnia magna*, and similar impact was observed on the juvenile larvae number per surviving adult upon PE-MP exposure. The combined exposure to deltamethrin and PE-MPs led to a synergistic detrimental effect on brood number and survival in this species (Felten et al., 2020). However, addition of the insecticide and endocrine disruptor dichlorodiphenyltrichloroethane (DTT) (Kelce et al., 1995) to PE-MPs had neither additive nor synergistic effect on the larval yield of inland silversides (*Menidia beryllina*).

Sex-specific differences in sensitivity towards MP-pollutant mixtures have also been observed. For example, in the female Japanese medaka (*Oryzias latipes*), a decrease in estrogen receptor (ERα) expression, and expression of the egg proteins, vitellogenin and choriogenin was recorded after 2-months dietary exposure to virgin or marine-weathered PE-MPs (Rochman et al., 2014), indicating possible adverse effects on oogenesis (Murata et al., 1997). However, in the male Japanese medaka, only virgin PE-MPs influenced gene expression, and marine-weathered PE-MPs did not cause significant alterations in the measured outputs (Andrady, 2011; Rochman et al., 2014), suggesting sexual dimorphism in this response. When the medaka was exposed to UV-treated or marine-weathered MPs at larvae stage, it showed greater induction of vitellogenin expression, an *in vivo* biomarker of estrogen action linked to reproductive effects, than what was measured in larvae exposed to virgin MPs. This suggests that early developmental stages of marine species may be more sensitive to a combination of MPs and their leached EDCs, than to MPs alone, consistent with the long-lasting impact of EDCs alone in early development of animals (Patisaul and Adewale, 2009). This could indicate that timing and length of MP-pollutant treatment would be crucial in determining impacts of these relevant to environmental exposure. The synergistic effects of MPs/NPs and their pollutants might be the consequence of enhanced cellular uptake of the particles, as shown in mouse cell culture experiments performed with weathered MPs (Ramsperger et al., 2020). Interestingly, MPs and NPs could also act antagonistically with persistent organic pollutants, by absorbing and therefore decreasing bioavailability of EDCs. For instance, exposing the crustacean *Gammarus roeseli* to MPs mixed with the EDC phenanthrene led to less detrimental effects than observed by exposure to phenanthrene alone (Bartonitz et al., 2020).

### Systematic Analysis Under Strictly Defined Experimental Conditions Are Vital to Determine Reproductive Effects of MPs and NPs

It should be noted that there are a number of studies, mostly performed on aquatic species, where there were no negative effects observed on reproduction upon MP/NP exposure. In *Daphnia pulex*, NPs caused no difference in the offspring number/clutch or female and the number of clutches in exposure during F0 or F1 generations (Liu et al., 2020b). In another study, *Daphnia magna* exposure to a mix of NPs and MPs showed no reproductive effects despite uptake of these plastics (Rist et al., 2017). In quagga mussels (*Dreissena rostriformis*), MP had no effect on reproduction perhaps due to an acute and short (24 h) exposure (Pedersen et al., 2020). Similarly, exposure of MP in *Danio rerio* (zebrafish) for a short period (2 days) had no effect on egg fertilization (Pitt et al., 2018). In some cases, such as in the blackworm, ingested PE-MPs over a longer, 28-day exposure increased ROS and decreased aerobic energy production but did not alter reproduction (Silva et al., 2021). In two studies, PS-MP exposure in Java and Japanese medaka over 21 days (Assas et al., 2020) or in *Daphnia magna* over 100 days (Kelpsiene et al., 2020) caused no reproductive defects. It is possible that the used MP doses in these experiments, lying in the lower end of environmental concentrations (i.e., 10^7^ particles/l equivalent to 44 μg/L for the medaka and 0.32 mg/L for *Daphnia magna*), were too low to induce overt defects in the reproductive function of these animals. In one case, MPs could be used as a substrate for egg deposition which led to an increase in the numbers of water strider adults and juveniles in the North Pacific (Goldstein et al., 2012; Majer et al., 2012). It is therefore important to use a range of concentrations of plastics over both acute and chronic durations in a systematic way to test toxicity.

## *C. elegans* as a Model for Comparative Studies of Plastics-Induced Reproductive Toxicity

From the studies considered above, it is clear that there are not enough systematically performed comparative analysis that assess the impact of various shapes, types, and sizes of MPs/NPs as well as sex or developmental stage at exposure on reproduction. In addition, few studies have compared the adverse effects of virgin plastic particles to plastic particles carrying pollutants, such as EDCs (Eder et al., 2021), due to the complexity of chemical mixtures found on MPs and NPs (Rochman et al., 2015; Bhagat et al., 2021). The nematode *C. elegans* model has several advantages that this research area could benefit from, in particular its potential to serve as a high throughput screening system, due to its small size, short lifespan, completely sequenced genome and transparent body. *C. elegans* has been extensively used in environmental toxicology research since it is sensitive to multiple environmental toxins, including organic pollutants and nanomaterials (Leung et al., 2008; Zhao et al., 2013; Jung et al., 2015). *C. elegans* may even be a more sensitive indicator of toxicity than other model organisms since they show significant reproductive disruption in response to lower concentrations of drugs or MPs when compared to other organisms (Zhang et al., 2019; Yu et al., 2020b). Toxicology screens performed in *C. elegans* show good correlation with toxicity assays in the classical vertebrate models (Hunt, 2017). Moreover, endpoints in *C. elegans* are similar to that examined in vertebrates. For example, MP and NP intake by *C. elegans* is linked to shorter lifespan, decreased survival rate, decreased progeny number, decreased body size, altered motility, and increased oxidative stress (Leung et al., 2008; Boyd et al., 2010; Zhao et al., 2013). Therefore, *C. elegans* provides a cost-effective promising model for testing varying types and sizes of plastics, and the combination of these and chemically complex pollutant mixtures. *C. elegans* offers high-throughput, whole animal screening assays that can be performed under controlled exposure conditions (Wittkowski et al., 2019), providing high level of reproducibility due to widely established standardized protocols. This is particularly important when comparing effects that may occur when many pollutants act synergistically to the impact of pollutants acting alone. In contrast to many *in vitro* cellular systems or more expensive rodent models with longer lifespans, high-throughput *C. elegans* toxicology assays using reporter genes readily expressed in worms can quickly assess the reproductive and endocrine response of the whole living, and metabolically active animal (Boyd et al., 2012; Boyd et al., 2016; Harlow et al., 2016). Results obtained in the *C. elegans* model could perhaps be translated to humans, since 83% of the *C. elegans* proteome has human orthologues (Lai et al., 2000).

The strong conservation of gene/protein structure and function, and molecular pathways between humans and *C. elegans* as well as the ease of gene deletion in worms makes the worm an attractive candidate to investigate the impact of environmental pollutants on organismal reproductive outputs and link these outputs to signaling pathways. However, it must be noted that *C. elegans* requires higher concentration of the chemicals to note a similar effect to that observed in rodents or in cell culture, due to their robust cuticle that forms a barrier to chemical uptake (Leung et al., 2008; Xiong et al., 2017; Wittkowski et al., 2019).

## Impairment in Gonadal Integrity and Gamete Quality Could Give Rise to MP/NP-Induced Reproductive Defects

Exposure to MPs/NPs was widely reported to alter gonadal morphology and decrease gamete number and quality in both sexes of aquatic and terrestrial species (Supplementary Tables S1, S2). Following exposure to PS-MPs, the pacific oyster showed significant decrease in sperm velocity similar to that of observed in MP-exposed male mice (Sussarellu et al., 2016; Xie et al., 2020). This may lower the ability of sperm to fertilize oocytes as lower sperm motility has been linked to decreased success in fertilization (Malo et al., 2006). What are the mechanisms underlying lower sperm quality? MPs affect gonad morphology by increasing cell death or apoptosis. MPs accumulate in the testes of mice (Jin et al., 2021) and rats (Li et al., 2021b) and disrupt the arrangement of the spermatid cells in the testicular seminiferous tubules (Hou et al., 2021a) leading to apoptosis of spermatogenic cells (Li et al., 2021b). These cells show pyknosis, nucleus rupture, and cell detachment upon MP-exposure, with widespread dose-dependent apoptosis in the testicular tissue. Similarly, male marine medaka (*Oryzias* melastigma) testes showed clear histological changes after MP exposure, with an increase in the interstitial tissue and disordered seminiferous lobules (Wang et al., 2019b). In mice and rats, MPs caused disruption of the blood testis barrier (BTB), with downregulation of the expression of associated junction proteins (Li et al., 2021b; Jin et al., 2021). Therefore, MP-driven direct testicular injury impedes spermatogenesis and decreases fertility in many species. Exposure to MPs also alters a testicular immune response, with increased expression of inflammatory factors and cytokines, suggesting increased testicular inflammation which may in turn also drive apoptosis and disruption of gonadal morphology. Due to MP accumulation, Nuclear factor-κB (NF-κB) was activated initiating apoptosis of the affected cells (Hou et al., 2021a). At higher MP concentrations, in male marine medaka there was dissolution of the basal membrane and spermatocytes became disorganized, perhaps due to the upregulation of chronic inflammation and oxidative stress (Wang et al., 2019b). Hence, in males of many species, a combination of an increase in apoptosis, oxidative stress and inflammation upon MP exposure appear to be instrumental in the detrimental changes to gonadal morphology and sperm quality.

Parameters that are used as a predictor of oocyte quality, such as number and diameter of oocytes, were significantly lower in MP treated female mice than in unexposed females (Sussarellu et al., 2016) (Supplementary Table S1). MPs entered the ovary of rats and decreased the volume of growing follicles when compared to the control animals (An et al., 2021b). Similarly, oysters exposed to MPs for 2 months showed a significant decrease in oocyte diameter and number (Sussarellu et al., 2016). As larger oocytes positively correlate with larval survival and growth, these studies suggest that MP exposure decreases the viability of the oocytes (Baynes and Howell, 1996). Consistent with this, in PS-exposed oyster females larval yield decreased compared to controls, suggesting that MPs cause low quality oocytes which in turn produce less larvae (Sussarellu et al., 2016). Additionally, female ovaries in the marine medaka had a lower number of mature spawning follicles and an increase in early vitellogenic oocytes in response to MP exposure. An MP-caused decrease in estrogen levels could be responsible for the impaired oogenesis with smaller oocytes (Wang et al., 2019b) and lead to delayed ovarian development in the fish (Bourguiba et al., 2003; Wang et al., 2019b). Similarly to its effect in testes, MPs caused apoptosis in the ovary and triggered oxidative stress, causing downregulation of Bcl-2 and upregulation of Bax in the granulosa cells. This can have an impact on female fertility as MP-triggered apoptosis may effectively decrease the available ovarian cells for oocyte development (Johnson et al., 2004; Johnson et al., 2005; Dunlop et al., 2014).

## The Effects of Microplastics and Nanoplastics on Reproductive Behaviors

Reproductive behavior is a useful output since it is ethologically relevant, possible to observe directly and reflective of both alterations in the brain and in the whole animal. It is one of the most sensitive indicators of toxin exposure of the central nervous system (Melvin and Wilson, 2013), as it can be observed using sublethal concentrations of the relevant toxins.

The EDCs’ effect on behavior was extensively investigated *in vivo*, including on behaviors that pertain to anxiety, feeding behavior or cognition (Frye et al., 2012; Palanza et al., 1999). Some studies also investigated exploration, aversion to novelty, partner preference and social interaction (Gillera et al., 2020; Krentzel et al., 2021). The impact of plastic particles on behavior is much less established, with only few studies reporting altered predator-prey interactions and hiding responses, decreased motility or changes to social interactions upon MP exposure of fish (Sarasamma et al., 2020; de Sá et al., 2015; Chagas et al., 2021; Wen et al., 2018; Yin et al., 2018; Chen et al., 2020b; Santos et al., 2021) or crustaceans (Gambardella et al., 2017; Tosetto et al., 2016b; Rehse et al., 2016; Suwaki et al., 2020; Bai et al., 2021). Given the reproductive deficit seen with MPs/NPs and the alterations seen in sex steroid hormone levels (as discussed below in *MPs/NPs Alter Nuclear Hormone Signaling and Biotransformation*), an interesting question is whether and to what extent MPs and NPs disturb related complex social behaviors, such as sexual behavior. The process of extensively studied vertebrate sexual behavior can be split into anticipatory and consummatory elements (Table 1), which are regulated by sensory systems, reward circuits and hormone signaling (particularly estrogen and androgens) in a sexually dimorphic manner in the male and female brain (Pfaus et al., 1990; Agmo et al., 2008; Swaney et al., 2012; Rebuli and Patisaul, 2016). Estrogens and androgens signal by binding nuclear hormone receptors i.e. the estrogen receptor (ER) and androgen receptor (AR), respectively. These receptors play a critical role in sexual differentiation of the brain *in utero*, to give rise to sexually dimorphic neural circuitry that drives reproductive behaviors in adulthood (McCarthy and Arnold, 2011). When signaling by the ER and AR are disrupted, alterations in sexually dimorphic behaviors are seen. Therefore adult behavioral “readouts” such as sex behavior in rodents have often been used to showcase the effect of prenatal exposure to low-dose EDCs (Patisaul and Adewale, 2009) that disrupt nuclear hormone receptor signaling, particularly if the exposure occurs during a critical prenatal or perinatal window. The potential interaction of MPs and NPs with nuclear hormone receptor signaling, such as regulated by ER and AR, is a possible entry point where plastic particles could affect a repertoire of complex reproductive behaviors in adulthood or via acting during development. Furthermore, as MPs and NPs show neurotoxic effects (Prüst et al., 2020), it is possible that disturbing neuronal circuits of reproductive/sex behaviors also contribute to decreased fertility and reproduction observed in animals. The emerging evidence supporting these theories are presented in the following sections.

**TABLE 1:** | Comparison of male sexual behavior steps in *C. elegans*, rats, and Japanese quail.

Steps	*C. elegans*	Rat	Japanese quail
1	Contact	Search and Contact	Search and Contact/Head grab
2	Scanning	Rooting	Attempted mounting
3	Turning		
4	Vulva location	Mounting	
5	Prodding	Mounting with thrusting	Successful mount
6	Spicule insertion	Intromission during mounting	Cloacal apposition
7	Ejaculation	Ejaculation	Sperm transfer

Mating behavior can be divided into analogous components (anticipatory or consummatory) for each species shown. C. elegans mating is described by 7 sub-behaviors, the rat mating is characterized by 6 sub-behaviors, and in the Japanese quail there are 5 steps described. These model organisms all begin mating behavior with searching and contacting the female/hermaphrodite at any place of the body. C. elegans and rat males then engage in a search for the vulva of the mate either through scanning and turning (C. elegans) or rooting (a form of chemo-investigation in rats). Upon location of the vulva, all three species begin mounting/prodding to locate the vulva precisely. Once this has been achieved, they position their sexual organs in order to aid ejaculation into the mate (Hull and Dominguez, 2007; Barr, 2014; dkins-Regan, 2014).

### The Impact of MPs/NPs on Sexual Behaviors

Sexual motivation is the first step of reproductive behavior and is part of the anticipatory component. As mating is key to species survival, animals are naturally motivated to perform this behavior, and this is intrinsically rewarded by the release of dopamine (Melis and Argiolas, 1995; Wise, 2004; Hull and Dominguez, 2007; Udupa and Chen, 2016). The dopaminergic meso (cortico)limbic system regulates the motivation for female sexual behavior and this circuit is regulated by estrogen signaling via the ER (Meisel and Mullins, 2006; Micevych and Meisel, 2017; Sanna et al., 2020).

When the effect of MPs on reproduction was examined after exposure to high levels of plastic particles, the planktonic crustacean *Daphnia magna* showed increased inter-brood periods and decreased average brood production, suggesting they had decreased motivation for reproduction (Ogonowski et al., 2016). In the zebra mussel (*Dreissena polymorpha*) exposure to different sizes of virgin PS-MP for 6 days increased dopamine levels (Magni et al., 2018), which could alter the motivation for reproduction. In echinoderms and bivalve molluscs, dopamine drives oogenesis (Khotimchenko, 1991). Since MP exposure causes dopaminergic neurotoxicity, a decrease in dopamine may influence oocyte quality in oysters (Hoelting et al., 2013). Similarly to what is observed in female mice, PS-MPs cause neurotoxicity in dopaminergic neurons and decreases dopamine levels in *C. elegans*. As dopamine also promotes egg-laying (Nagashima et al., 2016), PS-exposure leads to reduced egg-laying in the nematode model (Lei et al., 2018b). Supporting this, after exposure to and internalization of NPs, cultured human dopaminergic neurons developed neurospheres with increased oxidative stress (Hoelting et al., 2013). Given that MPs can alter the brain’s dopamine chemistry, possibly via ROS-induced apoptosis of dopaminergic neurons, the impact on reproductive motivation during and after exposure would be important to study.

Though the impact of MPs/NPs on consummatory components of sexual behavior has not yet been investigated, toxicity of EDCs that could leach from plastics have been widely studied, typically in rodents. For example, female rats and mice exposed to bisphenol A (BPA) as adults have increased plasma estrogen levels, which are linked to increased lordosis and reduced rejective behaviors during mating (Ribeiro et al., 2012; Wang et al., 2014). This may increase preference for less fit males (Patisaul and Adewale, 2009). In male rats, chronic adult exposure to BPA causes increased latencies to anticipatory and consummatory behaviors, including first mount, pelvic thrust, intromission, and ejaculation, and fewer intromissions when compared to the control animals (Picot et al., 2014). New, targeted studies assessing microplastic-induced alterations in nuclear-hormone-receptor signaling driving reproductive behaviors could shed light on their potential toxicity.

### Is *C. elegans* a Good Model to Explore the Impact of MPs/NPs on Reproductive Behaviors?

**FIGURE 1:**
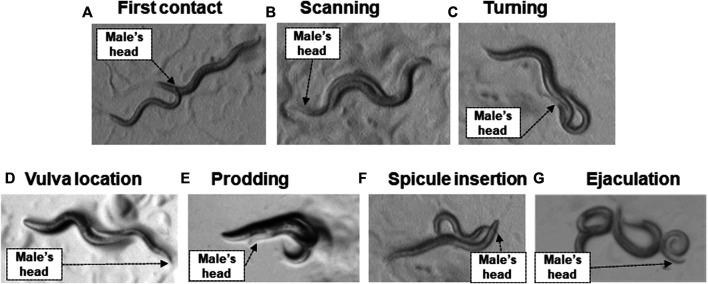
Steps in mating behavior of *C. elegans*. **(A)** First contact: the male contacts the hermaphrodite with its head. **(B)** Scanning: the male presses the ventral side of his tail against the hermaphrodite’s body then moves backwards while pressed against their body. **(C)** Turning: the male reaches the head or tail of the hermaphrodite and engages in turning behavior. The tail is in contact with the hermaphrodite. **(D)** Vulva location: the male locates the vulva and stops forward locomotion. The male’s tail is in contact with the hermaphrodite’s vulva. **(E)** Prodding: the male moves forward and backwards in small movements over the vulva to locate the vulva opening precisely. The tail is in contact with the hermaphrodite’s vulva. **(F)** Spicule insertion: the male inserts his spicules to open the lips of the vulva and allow sperm to flow freely into the uterus. The tail is in contact with the hermaphrodite’s vulva. **(G)** Ejaculation lasts for approximately 4 s and the spicules remain inserted for approximately a minute, however, due to scoring, ejaculation is determined as the time point when the male loses complete contact with the hermaphrodite as it is unclear when exactly ejaculation occurs (Barr and Garcia, 2006). The black arrow shows the position of the male’s head.

While *C. elegans* is a widely used model in investigating toxicology of MPs in eukaryotic multicellular organisms, our knowledge on the impact of MPs on reproductive behaviors even in this extensively studied species is limited. As in rodents, *C. elegans* male reproductive or sex behavior is a well-documented sequential mating behavior, with the males actively performing most of the sensory and motoric behaviors during the process (Figure 1) (Barr and Garcia, 2006). As in mammals, mating behaviors in *C. elegans* result from sexually dimorphic nervous systems. The hermaphrodite has 8 sex-specific neurons, whereas the male has 91 sex-specific motor, inter and sensory neurons, of which all but 4 are associated with the tail (Breedlove, 1986; Liu and Sternberg, 1995; Barr et al., 2018).

Experimental testing of reproductive capacity and mating behaviors so far has measured the time taken by the male to find the hermaphrodite, male spicule insertion, or measuring brood size to assess mating success (Barr, 2014). As in mammals, male mating effectivity in *C. elegans* decreases with age due to defects in mating execution rather than diminished sperm quality, suggesting that *C. elegans* infertility develops similarly to mammals (Chatterjee et al., 2013). Since there is a differential requirement for protein products across the mating sequence, the vulnerability of some behavioral mating stages to MPs/NPs may enable the identification of genes and pathways that are targeted by these pollutants. Due to the short lifecycle of *C. elegans*, which reaches reproductive stage in just 3 days post-hatching, along with the ease of obtaining replicates, and accuracy of behavior “scoring” parameters (Figure 1), *C. elegans* provide a cost-effective and rapid system for reproductive behavior testing when compared to rodents. Furthermore, *C. elegans* can provide insights into how early-life exposure to MPs/NPs might lead to deleterious consequences in later life. Due to its short lifespan, this nematode is ideal for studying the long term impacts of MP/NP exposure during the course of the whole lifetime (Litke et al., 2018). This is particularly relevant for the longer-lived human populations. Therefore, we propose that *C. elegans* is particularly suitable to investigate the impact of single environmental pollutants or combinations of these on the male mating behavior model (Figure 1) and resulting brood size in a longitudinal manner. In addition, investigating this will clarify if reproductive behavior and/or damage to the oocytes or sperms is the critical driver underlying decreased reproduction rate in animals upon exposure to plastics.

Dopamine signaling is well-conserved between vertebrates and *C. elegans*, and its function has been characterized in detail in the nematode model. *C. elegans* uses dopamine to react to environmental conditions, adjust its physiology and generate appropriate behaviors (McCloskey et al., 2017). The hermaphrodites have eight dopaminergic neurons that coordinates locomotion with egg-laying behavior (Cermak et al., 2020). The males have dopamine expression in the male ray sensory neurons, which enable them to respond to the presence of the hermaphrodite by moving towards and begin mating (Lints and Emmons, 1999), relevant to overall reproductive success of males. In *C. elegans* the expression of the dopaminergic neuron reporter dat-1pr::GFP shows decreased fluorescence upon exposure to polystyrene nanoplastics (PS-NP). This was also associated with decreased mitochondria function and increased oxidative stress (Liu et al., 2020c), suggesting dopaminergic specific toxicity upon PS-MP exposure. The exposure to UV-aged PS-MPs caused more severe dopaminergic defects than virgin MPs, probably due to the leaching of toxic materials (Chen et al., 2021a). Interestingly, PS-NP exposure in *C. elegans* causes significant increase in the expression level of intestinal *dop-1*, a gene encoding for a dopamine receptor (Qu et al., 2020a), further supporting the idea of MP-driven interference with the dopaminergic system in nematodes.

Changes of dopamine levels induced by MPs/NPs could affect a number of different dopamine-dependent behaviors that could be used to screen the toxic effects of MPs and their pollutants. For example, in *C. elegans* hermaphrodites touch response habituation is dependent on the availability of food. In the absence of food animals are habituated faster to the touch-triggered escape reflex than in the presence of food (Kindt et al., 2007). This response is regulated by dopamine (Kindt et al., 2007). Dopaminergic signaling is also required for the transition between locomotory gaits and slowing movement upon mechanosensation of food (Vidal-Gadea and Pierce-Shimomura, 2012; Sawin et al., 2000). Exposure to MPs decreased thrashing frequency when swimming in liquid and crawling speed on solid surface (Chen et al., 2021b) in a size and concentration dependent manner (Lei et al., 2018b). These behavioral assays could be used as high-throughput readouts upon exposure to plastic particles, prior to testing these in reproductive behavior assays (discussed in details in *C. elegans is a Promising Model to Investigate Molecular Pathways Mediating MP/NP-Induced Reproductive Toxicity*). Though reproductive motivation regulated by the dopamine system has not been studied in *C. elegans*, dopamine is involved in fine-tuning the activity of sensory-motor neurons and muscles during male copulation (Correa et al., 2012) and is a conserved candidate pathway. Hence, using dopamine synthesis or signaling worm mutants in these studies would be invaluable in understanding the contribution of dopaminergic neurons to reproductive behaviors as well as pinpointing possible action mechanisms for plastic pollutants.

## Signaling Pathways Involved in MP/NP-Induced Reproductive Toxicity

As seen above, MPs/NPs have been shown to induce reproductive toxicity in a wide range of aquatic and terrestrial animals. As might be expected, when MPs/NPs are ingested, their primary target site is the gut and stomach, as shown in the zebrafish (Sarasamma et al., 2020), pacific oyster (Sussarellu et al., 2016), mouse (Park et al., 2020), *Daphnia sp*. (De Felice et al., 2019), amphipods (Au et al., 2015), but eventually they could also spread to the liver, heart and brain (Pitt et al., 2018; Deng et al., 2021; Kwak and An, 2021). In *C. elegans*, NPs can be found in various tissues of the body, including the gut, pharynx, and vulva. Prolonged exposure to PS-NPs caused *acs-22* mutant worms to accumulate NPs in the gonad due to the dysfunctional intestinal barrier of this mutant (Kage-Nakadai et al., 2010). Gonad accumulation of NPs is also seen in wild-type nematodes albeit when exposed to 10-fold higher concentration of plastic particles (Qu et al., 2018). Within the cells of *C. elegans*, MPs/NPs have been found to localize in lysosomes (Chu et al., 2021). *C. elegans* is proven to be an ideal platform to study MP/NP accumulation due to its transparency, enabling fluorescently labelled MPs to be observed in the worm without need for dissection (Zhao et al., 2017). The mechanism by which MPs translocate from the primary sites to the secondary sites are unknown, but at cellular level endoctytosis or phagocytosis have been suggested as relevant cellular uptake mechanism (Rejman et al., 2004; Xia et al., 2008; Ekkapongpisit et al., 2012; Monti et al., 2015).

### MPs/NPs Alter Nuclear Hormone Signaling and Biotransformation

One commonly identified signaling pathway regulated by MPs and NPs appears to be steroid hormone signaling, whereby altered expression levels of steroidogenic enzymes impact levels of steroid hormones, leading to possible changes in feedback of the hypothalamic-pituitary gonadal axis (Vadakkadath Meethal and Atwood, 2005) (Supplementary Table S1). This could potentially alter social behaviors, with detrimental consequences for reproduction.

As might be expected, cytochrome P450 enzymes involved in xenobiotic transformations are upregulated upon MP/NP exposure in some species. Long term PS-exposure in the marine medaka (Wang et al., 2021) and *Daphnia pulex* (Liu et al., 2020b) induced the expression of P450 enzymes. The P450 enzyme families also contain steroidogenic synthesis enzymes, some of which are affected by MPs or MP-EDC mixtures (Supplementary Table S1). In the medaka, exposure to PS-MPs decreased the number of mature eggs in the female and sperm in the male, and increased several steroidogenic enzymes including STaR, the rate limiting enzymes of steroid hormone synthesis, as well as the 11β-HSD and aromatase enzymes required for cortisol and estrogen synthesis, respectively. This leads to a higher estrogen/testerosterone ratio, which in some cases, could be due to MP carried EDCs. For example, co-exposure of male medaka to ethinylestradiol (EE) and MPs synergistically decreased the level of GnRH in the brain and increased Cyp19a in the testis, suggesting that increased estrogen/testosterone ratio led to lower sperm counts (Wang et al., 2022). Higher levels of estrogen could perhaps negatively feedback the level of the pituitary reproductive hormones, follicle stimulating hormone (FSH) and lutenizing hormone (LH) (Wang et al., 2021). Similarly, in the adult male Nile tilapia, irregular sized NPs decreased sperm numbers, and this was correlated with lower levels of LH and FSH (Ismail et al., 2021). In male rat (Amereh et al., 2020; Ijaz et al., 2021) or mouse (Xie et al., 2020), there was decreased testosterone and decreased LH/FSH (in rat only) upon MP exposure, supporting demasculinisation of the hypothalamo-pituitary-gonadal (HPG) axis. This was associated with altered morphology and viability of sperms, with evidence for increased DNA damage and tissue lesions (Amereh et al., 2020). In the adult male zebrafish, reduced aggression and increased shoaling behavior combined with increased vitellogenin synthesis and aromatase expression (Sarasamma et al., 2020) is observed upon MP exposure, suggesting possible demasculinisation of behavior, possibly due to decreased dopamine levels. In some cases, sexual dimorphism is seen in the hormone response to PS-MPs/NPs. For example, in the marine medaka, unlike in the previous examples, steroidogenic enzymes, estrogen, testosterone, LH and FSH increased in males but decreased in females (Wang et al., 2019b), though the mechanism that links MP/NPs to altered steroidogenesis remains unknown.

### Increased ROS Contributes to Reproductive Dysfunction Upon MP/NP Exposure

Studies covering a wide range of aquatic and terrestrial animal species indicated that oxidative stress, due to increased ROS production in cells and tissues, is a major molecular event triggered by MP/NP exposure (Supplementary Tables S1, S2). Increased ROS levels and/or altered expression of oxidative stress defense system were detected upon MP/NP exposure in the marine rotifer *Brachionus* species, copepods (*Tigriopus japonicus*) (Xue et al., 2021; Yoon et al., 2021), *Daphnia* species (Liu et al., 2021b), green mussel (*Perna viridis*) (Hariharan et al., 2021), marine medaka (*Oryzias melastigma*), zebrafish (*Danio rero*) (Qiang and Cheng, 2021), Nile tilapia (*Oreochromis niloticus*) (Ismail et al., 2021), as well as terrestrial organism including *Eisenia sp.*, rat and mouse*.* In many instances, increased ROS content of cells and tissues was associated with reproductive defects, defined as decrease in viability, quality and number of oocytes or sperms, or decreased tissue/gonad integrity, as discussed above. ROS in turn induced apoptosis in the gonadal tissue leading to tissue damage, indicated by histopathological changes in reproductive tissues/gonads (Supplementary Table S1).

MPs/NPs could dysregulate the ROS scavenger system causing decreased gene expression or activity of these enzymes. Alternatively, higher ROS levels could deplete cellular ROS scavenging molecules, by increased use of these in battling oxidative stress. Increased oxidative stress in MP/NP exposed organisms would be expected to drive upregulation of the expression and/or activity of ROS-scavenging molecules and detoxification enzymes, such as superoxide dismutase (SOD), catalase (CAT), peroxidase, glutathione (GSH) and glutathione peroxidase, or glutathione-S-transferase. However, depending on the species, type of MP/NP used, exposure conditions and presence of pollutants on the plastics, a range of different responses were recorded. Studies found that expression levels or activity of SOD, CAT, and some components of the glutathione system decreased upon MP ingestion in worms (Huang et al., 2021), in zebrafish brain and liver (Umamaheswari et al., 2021), and the testes, ovaries and fertilized eggs of marine medaka (Wang et al., 2021), as well as in *Daphnia pulex* (Liu et al., 2020d), and *Perna viridis* (Hariharan et al., 2021). On the contrary, in the *Nile tilapia* nanoplastic particles of irregular shape caused increased serum levels of SOD and CAT, although these enzyme levels were not investigated in the reproductive organs of the fish, and the observed male reproductive deficit was most probably due to alterations in the serum luteinizing hormone and testosterone levels (Ismail et al., 2021). PS-MPs caused decreased levels of CAT and SOD in rats’ testes, which were associated with reduced sperm count, sperm motility and viability, probably due to the significant tissue damage seen in this tissue (Ijaz et al., 2021) (Supplementary Table S1). It has been suggested that MP-induced ROS could lead to DNA damage and defects in sperm cells, such as observed in earthworms (Huang et al., 2021), leading to decreased fertility. Interestingly, the availability of increased food supply in aquatic species could suppress the reproductive toxicity of PS-NPs (50 nm diameter spheres) (Yoon et al., 2021) or PE-MPs (10–22 μm) (Xue et al., 2021), affecting ROS levels or the function of endogenous ROS scavenging system altered by MPs and their pollutants. Therefore careful experimental design and replicable experimental conditions are vital for developing understanding of the real-life impact of MP/NP pollution on wild-life reproduction.

Increased ROS could be the consequence of enhanced ROS generation by mitochondria, as MP/NP exposure has been associated with altered mitochondria function. Decreased mitochondria membrane integrity was observed in MP treated rotifers (Jeong et al., 2016; Jeong et al., 2021), whereby increased oxidative stress and concurrent upregulation of the MAPK stress signaling pathway correlated with decreased fecundity (Jeong et al., 2016). Transcriptome analysis of PS-MP treated marine medaka also indicated the activation of MAPK pathways (Chen et al., 2020a) alongside reproductive deficits observed in the fish. Mice that ingested PS-MPs showed increased mitochondria membrane potential with increased ROS content and decreased GSH levels in oocytes, which were developing in inflamed ovaries, leading to overall decrease in reproduction (Liu et al., 2022b). A potential explanation for the observed mitochondrial dysfunction in various species upon MP/NP exposure could be lysosomal accumulation, and subsequent escape of the plastic particles to the cytosol via lysosomal rupture, which could lead to increased mitochondrial Ca^2+^ uptake and initiation of cell death, such as described in the murine RAW264.7 macrophage cells (Xia et al., 2008). Disruption of mitochondrial membrane potential was also observed in PS-MP-treated human epithelial colorectal adenocarcinoma cells (Caco-2) (Wu et al., 2019), suggesting a universal mechanism that could lead to increased ROS production and toxicity in animals upon plastic exposure. The resulting oxidative stress could cause damage to the DNA, lipids and proteins, ultimately leading to cell and tissue defects under sustained high ROS levels. Thus, increased ROS-induced cellular damage or mitochondria dysfunction-mediated cell death could be a probable explanation for reproductive tissue damage responsible to decreased fertility in animals.

It has been suggested that MP/NP-disruption of the blood-testis-barrier (BTB) leading to oxidative stress activates the p38/MAPK-Nrf2 pathway and induces apoptosis of spermatogenic cells, which could be responsible for the reduced reproductive capacity of PS-MP treated Wistar rats (Li et al., 2021c). PS-MP ingestion-induced elevated ROS in the testes of male Balb/c mice, which in turn activated the p38/MAPK stress signaling pathway, causing reproductive toxicity, seen by lower number and decreased motility of sperms, and increased rate of sperm deformity (Xie et al., 2020). Decreased BTB integrity following PS-MP feeding of male Balb/c mice was also linked to ROS-induced imbalance in mTORC1 and mTORC2 signaling, resulting in altered expression of actin cytoskeleton components, ultimately leading to spermatogenesis dysfunction (Wei et al., 2021). Transcriptome and protein expression data of PS-MP exposed mice also suggested an upregulation of the inflammatory signaling pathways, orchestrated by NF-κB. This was shown by increased expression of various inflammatory factors, along with downregulation of the phase II detoxification response regulator Nrf2 (Hou et al., 2021a; Jin et al., 2021), resulting in lower sperm quality in males. The ovaries of female rats fed with PS-MPs showed decreased GSH-Px, CAT, and SOD and increased MDA activities, while the number of growing follicles decreased with concurrent elevated levels of ovarian granulosa cell apoptosis and increased ovarian fibrosis (An et al., 2021b; Hou et al., 2021b). The latter process is thought to be enhanced by ROS initiated activation of Wnt/β-Catenin signaling pathway. Importantly, both ovarian apoptosis and fibrosis could contribute to the depletion of ovarian reserve capacity in female rats upon MP exposure.

Importantly, increased ROS content measured by *in vivo* dyes or fixative staining of NP/MP affected tissues/animals is broadly observed, even in studies which did not observe reproductive phenotypes (Silva et al., 2021). Furthermore, while reproductive deficits seen upon NP/MP exposure might require longer exposure times to plastic particles, cellular readouts of increased oxidative stress could be observed even after short (24 h) exposure time (Supplementary Tables S1, S2). Most studies reporting on reproductive deficits used polystyrene NPs (Supplementary Table S1), although increased ROS content, and consequently, upregulated oxidative stress responses seem to be the uniform response to NPs and MPs of various physicochemical properties. In support of the general role of ROS and subsequent activation of MAPK signaling pathway in mediating reproductive deficits of MPs/NPs, few studies have shown that supplementing the MP/NP-treated experimental models with antioxidants, such as N-acetylcysteine (NAC), or specific inhibitors of p38 MAPK, could lead to reversing or attenuating the deleterious effects of MPs/NPs on reproductive function (Xie et al., 2020; An et al., 2021b).

### *C. elegans* is a Promising Model to Investigate Molecular Pathways Mediating MP/NP-Induced Reproductive Toxicity

*C. elegans* was utilized widely to develop further understanding of the molecular events associated with MP/NP exposure (Supplementary Table S2). Taking advantage of tissue specific RNAi silencing of individual pathway components as well as utilizing readily available knockout mutants of virtually all genes of its genome, MPs and NPs have been shown to affect a range of signaling pathways in *C. elegans*. As shown by Shao et al. (Shao et al., 2019), PS-NP exposure (1 μg/L) caused downregulation of the insulin/IGF-1 signaling (IIS) pathway, decreasing expression levels of *daf-2* (insulin receptor gene) and increasing the expression of *daf-16*, encoding for the FoxO orthologue in *C. elegans*. DAF-16 is a key transcription factor integrating signals from various pathways, including IIS, AMPK pathway, JNK pathway, germline and TOR signaling, to modulate aging and stress, via shuttling from cytoplasm to nucleus (Sun et al., 2017). Decreased insulin signaling leads to altered expression of DAF-16 target genes involved in detoxification response (Freedman et al., 1993; Honda and Honda, 1999). Other studies showed the activation on ERK/MAPK or p38/MAPK in the neurons (Yang et al., 2021) and changes to the JNK/MAPK and the insulin signaling in the intestinal cells of *C. elegans* upon PS-NP exposure (Qu et al., 2020b; Liu et al., 2021d). These pathways are all hallmarks of oxidative stress response. NP exposure therefore has been linked to changes in the expression level and activity of central transcriptional regulators in *C. elegans* with well conserved functions and orthologues in mammals, many of which are similarly involved in the MP/NP response. Most of these transcription factors (TFs) are key mediators of longevity and stress response pathways, that orchestrate the organismal response to environmental stimuli and metabolic status of the cells (Denzel et al., 2019). Although some of these have been associated with regulation of reproduction, currently there is no mechanistic link established between PS-NP exposure, changes in TF signaling and altered reproduction and fertility in *C. elegans*. A recent study using UV-aged PS-MPs in *C. elegans* provided evidence of increased germline DNA damage and consequent increased apoptosis of germ cells as probable cause for declining reproduction rate in treated animals (Chen et al., 2022). ROS content of worms indeed increased in MP/NP-exposed hermaphrodites across numerous studies (Yu et al., 2021). The incurred oxidative stress could lead to increased germline apoptosis via activation of the MAPK pathway (Salinas et al., 2006).

Altered oxidative stress response is an effect that is not only universally observed in the treated animal populations, but can also be transmitted to the offspring of MP/NP treated mothers along with reproductive deficits, even in the absence of NP/MP in the offspring generation (Zhang et al., 2019; Liu et al., 2020b; Chen et al., 2021b; Liu et al., 2021c; Sobhani et al., 2021; Wang et al., 2021). Long-lasting impact of MPs/NPs on the oxidative stress defense pathways could potentially contribute to neurotoxicity detected in the offspring of plastic treated *C. elegans* mothers (Chen et al., 2021b; Liu et al., 2021c). In *C. elegans*, supplementation of sulphate modified PS-NPs in the food led to decreased reproductive rate in four subsequent offspring generation, probably due to higher proportion of aberrant chromosomes formed in the oocytes (Yu et al., 2021), which might be the result of oxidative damage to the DNA of plastic exposed mothers. Increased germline apoptosis was observed in multiple offspring generations of PS-NP exposed *C. elegans* hermaphrodites, suggesting enhanced germline depletion as explanation for the decreased brood size in offspring (Sun et al., 2021; Yu et al., 2021).

As for the impact of MPs and NPs on NHR signaling and consequent defects in reproductive behavior in the *C. elegans* model organism, limited research is available in the literature. Even though typical vertebrate hormones that act by binding NHRs have not been identified in *C. elegans*, this nematode has 284 nuclear hormone receptors, considerably more than humans and mice (Sluder et al., 1999; Taubert et al., 2011; Zhang et al., 2004). Additionally*, C. elegans* expresses an estrogen receptor (ER) orthologue, NHR-14, and an androgen receptor (AR) orthologue, NHR-69. Molecular docking simulations performed with NHR-14 or NHR-69 have shown that the endogenous hormone ligands of the human ER and AR, 17β-estradiol and testosterone (respectively), have similar binding activity to NHR-14 and NHR-69 as to the human receptors (Jeong et al., 2019). Therefore, these NHRs might be relevant to study in regard of deficits in reproductive behaviors and fertility upon MP and NP treatment, as disturbance in the level of estrogen and androgen receptor ligands have been widely observed upon MP/NP ingestion in other species (as discussed in *MPs/NPs Alter Nuclear Hormone Signaling and Biotransformation*, Supplementary Table S1) (Wang et al., 2019b; Amereh et al., 2020; Xie et al., 2020; Ijaz et al., 2021; Jin et al., 2021; Wang et al., 2021; Wang et al., 2022). A nuclear hormone receptor that has been investigated in relation to MPs in *C. elegans* is the sterol-sensing NHR-8, which regulates fat metabolism and stress responses (Jones et al., 2013; Magner et al., 2013). PS-NP exposure significantly increased the expression of *nhr-8* in wild-type worms, while loss-of-function *nhr-8* mutation increased sensitivity towards PS-NP toxicity, decreased locomotion and increased ROS production (Huanliang et al., 2020). PS-NP exposure also increased the expressions of the intestinal *linc-9* long non-coding RNA, which targets the nuclear hormone receptor NHR-77, linking nuclear hormone receptor signaling to MP/NP toxicity. *linc-9* RNAi treated *C. elegans* showed increased susceptibility to PS-NP induced defects, which was diminished upon RNAi knockdown of *nhr-77*, indicating a functional role for NHR-77 in PS-NP toxicity (Zhao et al., 2021). This implies that MPs and NPs could potentially intersect NHR signaling pathways in worms, even without the additive effect of carried EDCs. Uncovering the MP/NP-driven disruption of NHR signaling in *C. elegans* may also give us clues as to what potential metabolic defects could be expected in animals due to increased plastic pollution in the environment and in the food chain, and what implication for reproductive behaviors these might have. This will allow us to generate new models of MP/NP action for behaviors and cellular endpoints that might be common to most organisms and for those that are specific for higher organisms.

Altogether, these studies have shown that 1) *C. elegans* responds to MP/NP toxic insult with altering a set of conserved signaling pathways, including oxidative stress-MAPK, IIS or JNK pathways, as observed in other animals; and 2) nuclear hormone signaling is probably one conserved target of MPs and NPs across species. This provides us with an excellent opportunity to further develop the *C. elegans* model for high-throughput screening to unravel the mechanistic links of MPs/NPs and reproductive toxicity.

## Transgenerational Reproductive Effects of MPs and NPs

An emerging pattern seen now is that MPs/NPs and even EDCs seem to give rise to transgenerational effects impacting one or more generations of offspring of exposed animals (Zhou et al., 2020) (Supplementary Tables S1, S2). Therefore, the large quantities of MPs and NPs accumulated in the environment and in the food chain globally will continue to deliver adverse effects for a long time, impacting many future generations. Transgenerational impacts of environmental pollutants have also been acknowledged as critical contributors of many disease mechanisms (Skinner et al., 2010; Ho et al., 2012; Nilsson et al., 2018). MPs and NPs cause developmental and reproductive dysfunction in progeny of several MP-treated aquatic species, including *Daphnia magna* (Martins and Guilhermino, 2018), zebrafish (Pitt et al., 2018) (Lei et al., 2018a) and the marine medaka (Wang et al., 2019b). The progeny of MP exposed *C. elegans* mothers were shown to contain MPs and had significant reduction in brood size, decreased locomotion, and increased level of intestinal ROS (Zhao et al., 2017). These transgenerational effects could be mediated by direct transfer of MPs and NPs to the developing oocytes or to the embryos by the mothers, such as seen in the zebrafish (Pitt et al., 2018). Conversely, intestinal accumulation of NPs in exposed mothers could cause brood size reduction in four subsequent generations of offspring with no obvious accumulation of NPs observed in the germline or gonad of mothers, suggesting maternal effect of reproductive toxicity (Yu et al., 2021). Indeed, MP exposure could lead to changes in the epigenetic marks of the genome in fruit fly (*D. melanogaster*) (Zhang et al., 2020a).

Transgenerational effects have been well documented in *C. elegans* and these are driven by conserved molecular mechanisms involved in epigenetic regulation. MP/NP exposure could exert its effect through maternal epigenetic changes as suggested by Yu et al. (Zhao et al., 2017), who demonstrated that maternal NP exposure led to altered expression of histone methyl transferase genes and hypomethylation of the *ced-3* promoter region, encoding for the caspase 3 orthologue involved in apoptosis. This led to decreased brood size due to increased germline apoptosis in several offspring generations. Pointing to the potential of the nematode model for investigating transgenerational impacts of MPs on reproductive behaviors, we note here, that a behavioral response, namely the *C. elegans* pathogen avoidance behavior, has been transmitted across multiple generations upon exposure of the parental population to the pathogenic bacteria (Moore et al., 2019). Interestingly, deletion of the *C. elegans* putative testosterone receptor NHR-69 has recently been linked to loss of gentle touch response upon testosterone supplementation (Fischer et al., 2012). Remarkably, this impaired testosterone-mediated touch response has been transmitted to multiple generations via epigenetic alterations, a regulatory pathway that has also been implicated in vertebrate testosterone signaling (Baum, 2009; Murray et al., 2009). This provides further opportunities for the utilization of behaviors as an assay in *C. elegans* system to study the mechanisms underlying transgenerational reproductive toxicity of MPs and carried pollutants (Baugh and Day, 2020).

## Conclusion–What can we Learn From *C. elegans* to Understand Mechanisms Underlying MP/NP Toxicity

As we discussed above, male and female reproductive defects upon MP/NP exposure are reported for many animal species (Andersson et al., 2008; D'Angelo and Meccariello, 2021; Blackburn and Green, 2021; Vo and Pham, 2021; Ji et al., 2021; Sharifinia et al., 2020). Although in recent years some mechanistic insights into MP/NP action have been uncovered, only few pathways have been directly linked to reproductive deficits and it is still unclear what the first, initiating steps are in MP/NP toxicity. We discussed the potential of oxidative stress and altered NHR signaling as common regulatory pathways targeted by MPs/NPs to cause reproductive dysfunction. Importantly, plastic polymers can directly cause tissue damage and apoptosis in the reproductive organs of animals, increase ROS production, interfere with hormone and nuclear hormone receptor levels, or alter energy status of cells. Interestingly, endocrine disrupting chemicals carried by NPs and MPs also cause similar alterations, including increased ROS and changes to hormone and NHR levels or activity. All of these pathways could be and partly have been explored in *C. elegans* (Supplementary Table S2) due to strong conservation of molecular pathways existing in the nematode model.

One consequence of MP/NP accumulation in the gut across species appears to be the alteration of gut microbiota. For example, in the marine medaka (Yan et al., 2020), the soil nematode *Enchytraeus crypticus* (Zhu et al., 2018) and in the soil springtail (*Folsomia candida*) (Ju et al., 2019), ingestion of PS-MPs or PE-MPs decreased the diversity of gut microbiota. A recent study in the springtail has suggested that gut microbiota dysbiosis caused by MP exposure could explain the observed decrease in reproduction rate, as healthy gut microbiome is essential for proper nutrient supply and immune protection for springtails (Ju et al., 2019). Germ-free *Drosophila* with no microbiota had lower aggression levels and lower reproductive fitness due to alterations in octopamine signaling (Jia et al., 2021), demonstrating that microbiota-influenced social behaviors can cause reproductive deficits. Furthermore, as microbiota can increase free estrogen levels of the host by deconjugation (Littman and Pamer, 2011), reduction of gut microbiota diversity decreases this process with a negative impact on host fertility (Plottel and Blaser, 2011). Gut microbiota also produce many bioactive small molecules that may act as nuclear hormone receptor ligands (Donia and Fischbach, 2015), directly interacting with the NHR signaling pathways of the host (Duszka and Wahli, 2018). Therefore, MP/NP-mediated interference with microbiota composition can be detrimental to host reproductive capacity. Being bacterivore species, this can be more easily explored in a *C. elegans* model where gut microbiota can be changed by feeding a defined single or a combinations of bacteria strains to worms (Zhang et al., 2017). Interestingly, in *C. elegans*, fecal microbiota transplants reversed oxidative stress by inducing GSH via the PMK/SKN-1 pathway, leading to attenuation of NP-mediated toxicity (Chu et al., 2021).

Reproductive behavior could also be utilized as an endpoint in *C. elegans* for investigating the behavioral deficits that decrease reproductive capacity upon MP/NP exposure. In the *C. elegans* model the whole neuronal connectome is mapped and changes in the dopaminergic neurons caused by MPs/NPs could provide a probe to understand how these circuits drive reproductive behavior. Given that a Pubmed search using the search terms (rodent AND (microplastics OR nanoplastics) AND behavior) resulted in only 11 studies (accessed on 25th January 2022) with most of these studies detailing the accumulation of MPs, it is clear that this is a field ripe for investigation, using any model and any behavior that is tractable to analyze. Since this behavior is sexually dimorphic, it could also point to a shift towards one sex and potential disturbance of the sex ratio upon MP/NP exposure. For example, in *Daphnia pulex* (Zhang et al., 2020b), increase in doublesex transcripts and lower energy reserve upon exposure to PS-NPs shifts the population to contain more males in a typically asexual population. This is a response to stressors to increase the rate of genetic recombination in the affected population (Mitchell et al., 2004). *C. elegans* populations show a similar increase in male populations in response to stressors and could be used to investigate how the shift in sex can occur to pinpoint molecular pathways that may be similar to other species (Morran et al., 2009).

We propose that the male *C. elegans* mating behavior is a model reproductive behavior that is ethologically relevant, reproducible, quick to analyze and can give information about genes and signaling pathways that are impacted by microplastics and nanoplastics. Given that MPs/NPs are almost always present with EDCs, these nematodes provide a promising platform with high-throughput potential to develop understanding of the reproductive effects of environmentally relevant pollutant-MP mixtures. We also emphasize that some of the genes targeted by MPs/NPs and leachates that disrupt the endocrine system will be nuclear hormone receptors and study of environmental pollutants in a simpler model has the potential to elucidate novel aspects of NHR signaling in biology.

## Supplementary Material

Supplementary Table S1 Toxic effects of MPs/NPs on reproduction and reproductive behaviors with potential action mechanisms uncovered. Green highlight indicates accumulation of cellular ROS, induced oxidative stress pathway as major mediator of toxicity observed in the study. Blue highlights indicate alterations in hormonal signaling, steroid hormone pathways as probable effector of MP/NP toxicity. For detailed literature review on model organisms in microplastics research the reader is suggested to read the publication from Prokic et al. (2021).

Supplementary Table S2 Toxic effects of MPs/NPs on reproduction and reproductive behaviors in *C. elegans* with potential action mechanisms uncovered. Green highlight indicates accumulation of cellular ROS, induced oxidative stress pathway as major mediator of toxicity observed in the study. Blue highlights indicate alterations in hormonal signaling, steroid hormone pathways as probable effector of MP/NP toxicity.
